# The Protective Properties of the Strawberry (*Fragaria ananassa*) against Carbon Tetrachloride-Induced Hepatotoxicity in Rats Mediated by Anti-Apoptotic and Upregulation of Antioxidant Genes Expression Effects

**DOI:** 10.3389/fphys.2016.00325

**Published:** 2016-08-05

**Authors:** Sherifa S. Hamed, Nouf A. AL-Yhya, Manal F. El-Khadragy, Ebtesam M. Al-Olayan, Reem A. Alajmi, Zeinab K. Hassan, Salwa B. Hassan, Ahmed E. Abdel Moneim

**Affiliations:** ^1^Department of Zoology, College of Science, King Saud UniversityRiyadh, Saudi Arabia; ^2^Department of Zoology, Faculty of Science, Alexandria UniversityAlexandria, Egypt; ^3^Chair Vaccines Research of Infectious Diseases, Faculty of Science, King Saud UniversityRiyadh, Saudi Arabia; ^4^Department of Zoology and Entomology, Faculty of Science, Helwan UniversityCairo, Egypt; ^5^Department of Cancer, National Cancer Institute, Cairo UniversityCairo, Egypt; ^6^College of Medicine, Princess Nora Bint Abdulrahman UniversityRiyadh, Saudi Arabia; ^7^Department of Clinical Pathology, College of Medicine, Fayoum UniversityFayoum, Egypt

**Keywords:** strawberry, carbon tetrachloride, oxidative stress, gene expression, fibrosis, liver

## Abstract

The strawberry (*Fragaria ananassa*) has been extensively used to treat a wide range of ailments in many cultures. The present study was aimed at evaluating the hepatoprotective effect of strawberry juice on experimentally induced liver injury in rats. To this end, rats were introperitoneally injected with carbon tetrachloride (CCl_4_) with or without strawberry juice supplementation for 12 weeks and the hepatoprotective effect of strawberry was assessed by measuring serum liver enzyme markers, hepatic tissue redox status and apoptotic markers with various techniques including biochemistry, ELISA, quantitative PCR assays and histochemistry. The hepatoprotective effect of the strawberry was evident by preventing CCl_4_-induced increase in liver enzymes levels. Determination of oxidative balance showed that strawberry treatment significantly blunted CCl_4_-induced increase in oxidative stress markers and decrease in enzymatic and non-enzymatic molecules in hepatic tissue. Furthermore, strawberry supplementation enhanced the anti-apoptotic protein, Bcl-2, and restrained the pro-apoptotic proteins Bax and caspase-3 with a marked reduction in collagen areas in hepatic tissue. These findings demonstrated that strawberry (*F. ananassa*) juice possessed antioxidant, anti-apoptotic and anti-fibrotic properties, probably mediated by the presence of polyphenols and flavonoids compounds.

## Introduction

The liver is the largest internal organ and the largest gland in the body; it plays an important role in complex metabolism and has numerous functions, including glycogen storage, lipid and protein synthesis, bile salt production, hormone production, and detoxification (Hampsey and Karnsakul, 2013). Liver injury may be induced by various pathological factors such as hepatic viruses and, chemical hepatotoxins; additionally, fatty livers lead to the down-regulation of many drug metabolizing enzymes (Grattagliano et al., 2009). Drug/chemical-induced liver injury, also known as toxic hepatopathy, is a major clinical problem (Chen et al., 2015). Impairment of the liver can not only affect growth and nutritional status, but may lead to acute, chronic, or end stage liver disease (Hampsey and Karnsakul, 2013) and subsequently influence the pharmacokinetic behaviors of many drugs (Verbeeck, 2008; Xie et al., 2010). Vitaglione et al. (2004) suggested that reactive oxygen species (ROS), including superoxide and hydroxyl radicals are known to play an important role in liver disease's pathology and progression and have also been associated with the intoxication by CCl_4_.

In recent years, considerable clinical and experimental evidences has shown that oxidative stress (caused by an imbalance between the oxidant and antioxidant systems of the body in favor of the oxidants) should be a major apoptotic stimulus in the different types of acute and chronic liver injury and hepatic fibrosis (Al-Olayan et al., 2014).

Hepatic fibrosis induced by CCl_4_ is associated with the exacerbation of lipid peroxidation and the depletion of antioxidant status (Fu et al., 2008). Accordingly, successful antioxidant interventions, which have attracted interest from investigators, offer insights into delaying or preventing the occurrence and development of hepatic fibrosis. Therefore, these interventions may be a potential therapeutic strategy for the prevention and treatment of hepatic fibrosis (Deng et al., 2012).

There is an intimate relationship between nutrition and the antioxidant defense system, as some exogenous low molecular weight antioxidants may be supplied by the diet. These two main systems of the antioxidant defense system act in coordination together, their levels regulate one another to avoid oxidative stress events (Masella et al., 2005). In the past few years, a considerably large group of molecules, widespread in plants, has come into focus.

In recent years, considerable clinical and experimental data has revealed that oxidative stress is caused by an imbalance between the oxidant and antioxidant systems within the body (Ghatak et al., 2011). There is a great deal of evidence indicating that natural substances from edible and medicinal plants exhibit strong antioxidant activity that could act against hepatic toxicity caused by various toxicants (Othman et al., 2014). One of those candidate plants is strawberry.

The strawberry (*Fragaria ananassa*) is a relevant source of bioactive compounds because of its high levels of vitamin C, folate, and phenolic constituents (Proteggente et al., 2002). The major class of phenolic compounds is represented by flavonoids (mainly anthocyanins, with flavonols serving as minor contributor), followed by hydrolyzable tannins (ellagitannins and gallotannins) and phenolic acids (hydroxybenzoic and hydroxycinnamic acids), with condensed tannins (proanthocyanidins) as minor constituents (Kahkonen et al., 2001; Aaby et al., 2005). Previous studies have shown that the strawberry that are rich in flavonoids, which are responsible for the antioxidant properties, as well as compounds isolated from the entire plant were promising in cancer chemopreventive therapy.

Berry seeds are a rich source of polyunsaturated fatty acids. These acids are not synthesized in the human body and have to be supplied through the diet (Pieszka et al., 2013). Moreover, the strawberry is a source of several other vitamins, such as thiamin, riboflavin, niacin, vitamin B6, vitamin K, vitamin A, and vitamin E. it has also been qualified as a good source of iodine, magnesium, copper, iron, and phosphorus (Giampieri et al., 2012). Thus, the present study aims to investigate the hepatoprotective role of strawberry (*F. ananassa*) juice against CCl_4_-induced liver toxicity in rats.

## Materials and methods

### Chemicals

Carbon tetrachloride (CCl_4_) and Tris-HCl buffer were purchased from Sigma Aldrich (St. Louis, MO, USA). Perchloric acid, thiobarbituric acid (TBA), and trichloroacetic acid (TCA) were purchased from Merck. All other chemicals and reagents used in this study were of analytical grade. Double-distilled water was used as the solvent.

### Animals

Forty adult male Wistar albino rats weighing 250–300 gm (10–13 weeks old) were obtained from The Animal House of King Saud University. The animals were kept in wire bottomed cages in a room under standard condition of illumination with a 12-h light-dark cycle at 25 ± 1°C for 1 week until the beginning of treatment. They were provided with tap water and a balanced diet *ad libitum*. All animals have received human care in compliance with the state authorities following the National Program for Science and Technology of Faculty of Science, King Saud University rules for animal protection.

### Plant material

Fresh strawberry (*F. ananassa*) were collected from the market of Saudi Arabia, Riyadh in the months of March–May, 2015. The plant material was authenticated by the Botany Department, Faculty of Science, King Saud University, on the basis of taxonomic characters and by direct comparison with the herbarium specimens available at the herbarium of the Botany Department.

### Strawberry juice preparation

Ten kilogram of strawberry were washed its leaves removed. Juice was obtained using a commercial blender (Philips, China), filtrated with a Buchner funnel and immediately diluted with distilled water to volume of 1:3 and stored at −20°C for no longer than 2 months (Faria et al., 2007).

### HPLC analysis

Analysis of the strawberry juice was performed using a Perkin Elmer Series 200 liquid chromatography (PerkinElmer, USA). The HPLC system was equipped with an autosampler, a C18 column from Teknokroma (Barcelona, Spain) and a photo diode array detector (PDA; model Series 200) The mobile phase was composed of water and methanol with the gradient elution system at a flow rate of 0.8 ml/min. Twenty-five micro liter was injected after filtration through a 0.22 μm PVDF membrane. The detection UV wavelength was set at 280 nm. The column temperature was ambient.

### Experimental protocol

To study the protective effects of the strawberry on carbon tetrachloride mediated hepatotoxicity, 40 adult male rats were randomly allocated to four groups of 10 rats each. Con (Control group) served as the control and received 300 μl of saline via intraperitoneal (i.p.) injection each week. CCl_4_ (CCl_4_ group) received weekly i.p. injection of 2 ml CCl_4_/kg body weight for 10 weeks as described by Abdel Moneim and El-Khadragy (2013). Strb (Strawberry group) received strawberry juice supplied in dark water bottles and renewed every 2 days. Animals belonging to Strb+CCl_4_ (Strawberry and CCl_4_ group) received strawberry juice for 2 weeks prior to and during the 10 weeks of i.p. injection with CCl_4_ treatment at a dose of 2 ml CCl_4_/kg body weight.

One week following the last i.p. injection of CCl_4_, the animals of all groups were killed by cervical dislocation and blood samples were collected. Followed by standing for 15 min at room temperature and then centrifuged at 1000 g for 10 min at 4°C to separate serum and stored at −70°C. All the animals were dissected, and the liver was excised, washed in normal saline and stored at −80°C for biochemical estimations.

### Liver function test

The amounts of alanine aminotransferase (ALT) and aspartate aminotransferase (AST) in the serum were estimated according to the method of Reitman and Frankel (1957), by measuring the amount of pyruvate or oxaloacetate produced by reaction with 2,4-dinitrophenylhydrazine spectrophotometrically at 546 nm.

### Oxidative stress markers

Homogenates of the liver were prepared in 50 mM Tris-HCl and 300 mM sucrose to determine lipid peroxidation (LPO) as thiobarbituric acid reactive substances (TBARS) using the method of Ohkawa et al. (1979). Additionally, the homogenates were used to determine nitrite/nitrate (nitric oxide; NO) (Green et al., 1982) and glutathione (Ellman, 1959).

### Enzymatic antioxidant status

Homogenates of the liver were used in the determination of superoxide dismutase (SOD) based on the inhibition of the formation of NADH-phenazine methosulphate-nitroblue tetrazolium formazon (Nishikimi et al., 1972), catalase (CAT) by using hydrogen peroxide as standard substrate (Aebi, 1984) and glutathione peroxidase (GPx) by measuring the reduction in NADPH absorbance monitored at 340 nm (Paglia and Valentine, 1967).

### Histopathological examination

Tissue samples were fixed in 10% neutral formalin for 24 h and paraffin blocks were routinely processed for light microscopy. Slices of 4–5 μm were obtained from the prepared blocks and stained with hematoxylin and eosin as well as Masson's trichrome for hepatic fibrosis. The preparations obtained were visualized using a Nikon microscope at a magnification of 400×.

### Determination of apoptotic markers in liver tissue

Liver homogenates were made in lysis buffer and analyzed using a colorimetric caspase-3 assay kit (Product number: CASP3C; Sigma-Aldrich Co. USA) according to the manufacturer's instructions. The concentrations of caspase-3 in liver lysates were calculated with the help of the calibration curve generated using known amounts of standards. Bcl-2 (Cat. No. LS-F10920) and Bax (Cat. No. LS-F5064) levels were measured in the liver tissue lysates by ELISA kits, (LifeSpan BioSciences, Inc., Seattle, WA, USA). The procedure was performed according to instructions of manufacturer. Levels were expressed as ng/mg tissue protein.

### Real time PCR

The total RNA was isolated from the liver tissue using an RNeasy plus Minikit (Qiagen, Valencia, CA). One microgram of the total RNA and random primers were used for cDNA synthesis using the RevertAid H minus Reverse Transcriptase (Fermentas, Thermo Fisher Scientific Inc., Canada). For real time PCR analysis, the cDNA samples were run in triplicate and β-actin was used as a reference gene. Each PCR amplification included non-template controls and all reagents except for the cDNA. Real time PCR reactions were performed using Power SYBR Green (Life Technologies, CA) and were conducted on the Applied Biosystems 7500 Instrument. The typical thermal profile is 95°C for 3 min, followed by 40 cycles of 95°C for 15 s and 56°C for 30 s. After PCR amplification, the ΔCt was calculated by subtracting the β-actin Ct from each sample Ct. The method of Pfaffl was used for the data analysis (Pfaffl, 2001). The PCR primers for SOD, CAT and GPx genes were synthesized by Jena Bioscience GmbH (Jena, Germany). Primers were designed using the Primer-Blast program from NCBI. For a reference gene, β-actin was used. The primer sets used were as the following:

**β-Actin**: sense: 5′–GGCATCCTGACC CTGAAGTA-3′, antisense: 5′–GGGGTGTTGAAGGTC TCAAA-3′; **CAT**: sense: 5′–TCCGGGATCTTTTTA ACGCCATTG-3′, antisense: 5′–TCGAGCACGGTA GGGACAGTTCAC-3′; **SOD2**: sense: 5′–AGCTGC ACCACAGCAAGCAC-3′, antisense: 5′–TCCACCACC CTTAGGGCTCA-3′ and **GPx1**: sense: 5′–CGGTTT CCCGTGCAATCAGT-3′, antisense: 5′–ACACCG GGGACCAAATGATG-3′.

### Statistical analysis

Results were expressed as the mean ± standard error of the mean (SEM). Data for multiple variable comparisons were analyzed by one-way analysis of variance (ANOVA). For the determination of significance difference between groups, Duncan's test was used as a *post-hoc* test according to the Statistical Package for the Social Sciences (SPSS version 17.0).

## Results

### HPLC result

**Figure 1:**
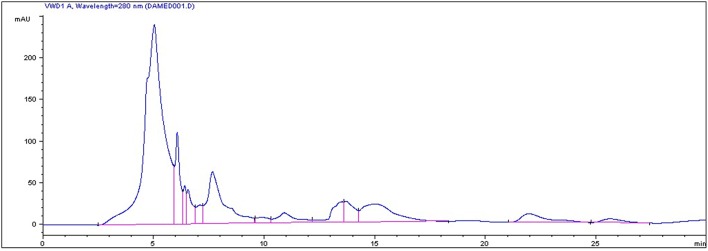
**HPLC chromatogram of strawberry (*Fragaria ananassa*) juice**.

In the current study, strawberry juice was subjected to HPLC analysis. HPLC experiment allowed for the identification of 18 compounds (Figure 1). The retention times and the fragment ion peaks are described in Table 1. Hydrolyzable tannins (gallotannins) were among the main class of (poly)phenolics identified in the strawberry juice. The fruits yielded mainly gallic acid derivatives (as glucosides, galloylquinic acid, galloylshikimic acid, and gallotannins), some proanthocyanidins, quercetin derivatives, and ellagic acid derivatives. The anthocyanins were not abundant in these fruits, but were identified as delphinidin-3-galactoside, cyanidin-3-galactoside, cyanidin-3-glucoside, and cyanidin-3-arabinoside. In addition, a broad number of anthocyanins, non-colored flavonoids and phenolic acids were also recovered. Other phytochemicals, such as lignans, and different flavonoids belonging to the four subclasses of non-colored flavonoids (flavan-3-ols, flavonols, dihydrochalcones and flavanones) were detected. The detected flavan-3-ols was (+)-gallocatechin. Flavonols displayed by the presence of kaempferol, phlorizin and quercetin. Whereas, dihydrochalcones displayed the flavanones subclasse.

**Table 1:** **Identification of phenolic compounds of the strawberry fruits**.

**Peak**	**RT (min)**	**Identified compound**
1	3.62	Ascorbic acid
2	4.51	Procyanidin trimer
3	4.86	Pelargonidin-3-diglucoside
4	5.03	galloyl-bis-HHDP-glucose
5	6.63	Cyanidin-3-glucoside
6	6.40	Pelargonidin-3-rutinoside
7	6.56	Ferulic acid hexose derivative
8	7.09	p-Coumaryl hexose
9	7.66	Pelargonidin-3-rutinoside
10	8.10	Quercetin-3-glucuronide
11	8.90	Pelargonidin-3-malonylglucoside
12	9.86	Kaempferol-glucuronide
13	10.91	Caffeic acid
14	13.52	Procyanidin pentamer
15	15.02	Sanguiin H-6
16	15.60	Ellagic acid pentoside
17	21.96	penta-O-Galloylglucoside
18	25.62	Gallotannins

*Abbreviation: RT, retention time*.

### Biochemical results

#### Effect of CCl_4_ on liver function markers

**Figure 2:**
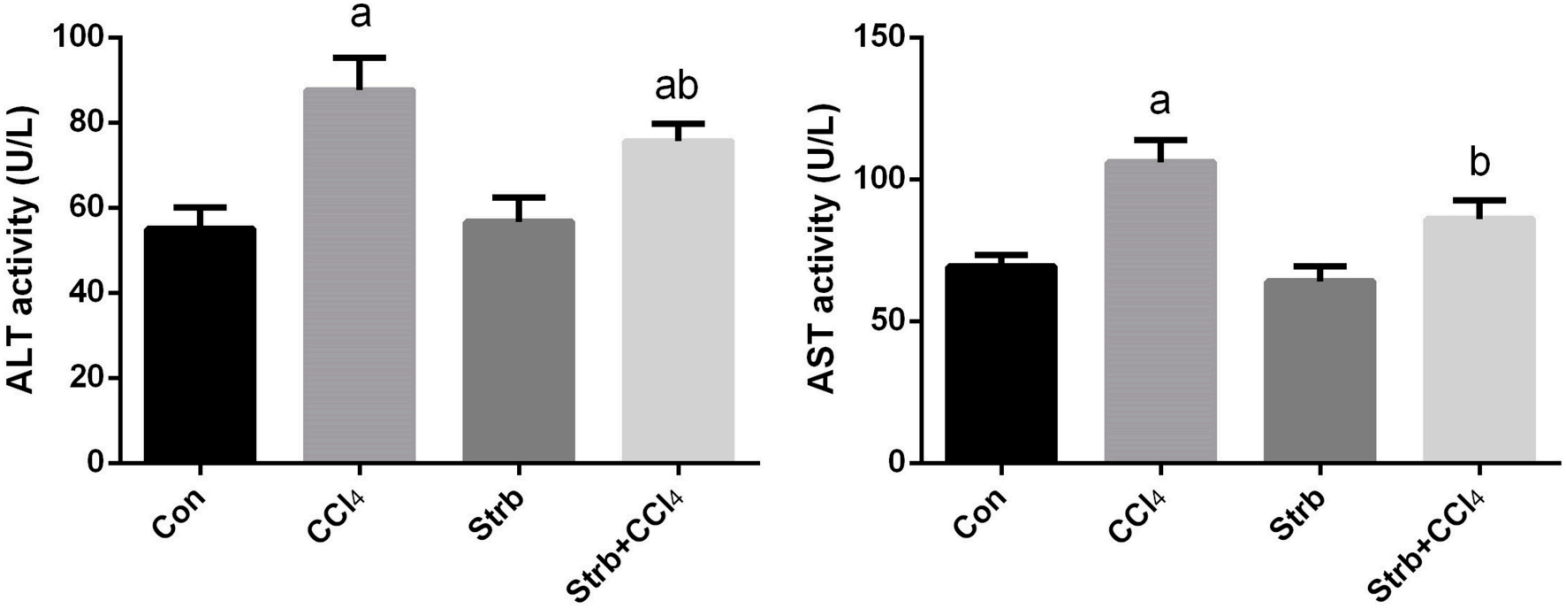
**Ameliorative effects of strawberry juice supplementation for 12 weeks on alanine aminotransferase (ALT) and aspartate aminotransferase (AST) in rats treated with CCl_4_ (2 ml CCl_4_\kg body weight) for 10 weeks**. Values are means ± SEM (*n* = 10). a: significant change at *p* < 0.05 with respect to the Control group. b: significant change at *p* < 0.05 with respect to the CCl_4_ group.

Transaminase enzymes are known as important markers of hepatocellular damage. In the current study, following CCl_4_ injection, the activity of serum ALT and AST was significantly (*p* < 0.05) elevated in comparison with the control rats (Figure 2). Pre-supplementation of rats with the strawberry juice restrained the liver function parameters from rising.

#### Effect of CCl_4_ on oxidative stress markers

**Figure 3:**
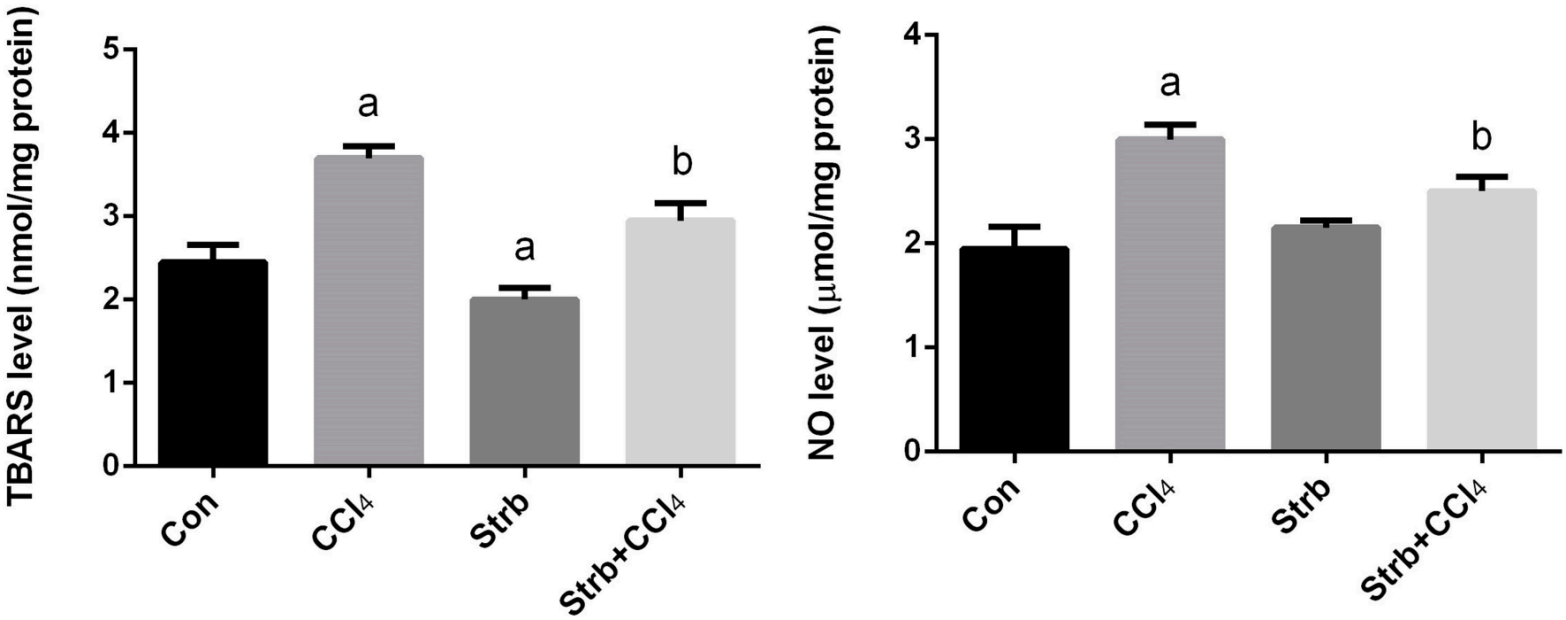
**Ameliorative effects of strawberry juice supplementation for 12 weeks on hepatic thiobarbituric acid reactive substances (TBARS) as a marker of lipid peroxidation and nitrite/nitrate as a marker of nitric oxide in rats treated with CCl_4_ (2 ml CCl_4_\kg body weight) for 10 weeks**. Values are means ± SEM (*n* = 10). a: significant change at *p* < 0.05 with respect to the Control group. b: significant change at *p* < 0.05 with respect to the CCl_4_ group.

Lipid peroxidation has been implicated in the pathogenesis of hepatic injury by the free radical derivatives of CCl_4_ and is responsible for both the cell membrane damage and consequent release of marker enzymes of hepatotoxicity. TBARS, a byproduct of lipid peroxidation, are widely used as a marker of lipid peroxidation. In addition to LPO, nitric oxide seems to play a major role in the pathogenesis of chronic liver disease and considered as a marker for oxidative stress. Furthermore, NO is also thought to be the cause of some complications associated with end-stage liver disease. To examine the effect of CCl_4_ on oxidative stress markers, TBARS and nitrite/nitrate levels in the liver homogenates of rats treated with CCl_4_ were measured and its levels were shown in Figure 3. CCl_4_ injection caused a significant (*p* < 0.05) increase in the levels of TBARS and nitrite/nitrate in liver compared to the control group. However, strawberry supplementation markedly attenuated TBARS and nitrite/nitrate levels in the CCl_4_ administered rats.

#### Effect of CCl_4_ on enzymatic and non-enzymatic antioxidant status

**Figure 4:**
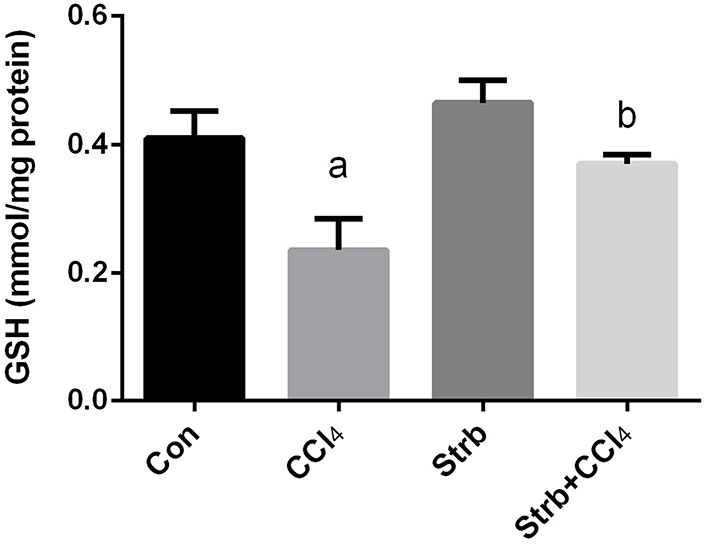
**Ameliorative effects of strawberry juice supplementation for 12 weeks on hepatic glutathione (GSH) content in rats treated with CCl_4_ (2 ml CCl_4_\kg body weight) for 10 weeks**. Values are means ± SEM (*n* = 10). a: significant change at *p* < 0.05 with respect to the Control group. b: significant change at *p* < 0.05 with respect to the CCl_4_ group.

The CCl_4_-induced oxidative stress was evident and indicated a significant reduction (*p* < 0.05) in the GSH contents of the liver tissue in CCl_4_-treated rats when compared to the control group. This reduction in the GSH contents was attenuated by the strawberry juice supplementation as shown in Figure 4.

**Figure 5:**
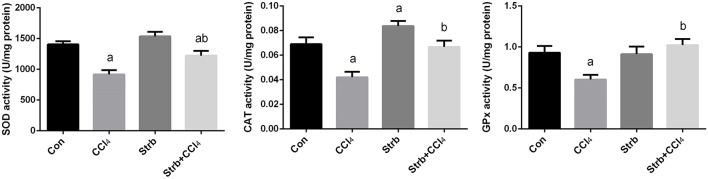
**Ameliorative effects of strawberry juice supplementation for 12 weeks on hepatic superoxide dismutase (SOD), catalase (CAT) and glutathione peroxidase (GPx) activities in rats treated with CCl_4_ (2 ml CCl_4_\kg body weight) for 10 weeks**. Values are means ± SEM (*n* = 10). a: significant change at *p* < 0.05 with respect to the Control group. b: significant change at *p* < 0.05 with respect to the CCl_4_ group.

**Figure 6:**
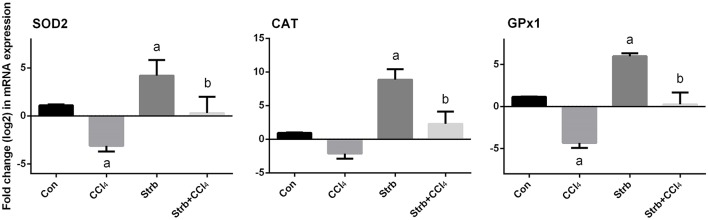
**Effect of strawberry juice supplementation for 12 weeks on hepatic mRNA expression of candidate genes in experimental groups**. Results (mean ± SEM of three assays) were normalized to β*-actin* RNA level and are shown as fold induction (in log2 scale) relative to the mRNA level in the control. SOD2, superoxide dismutase; CAT, Catalase; GPx1, glutathione peroxidase. a: significant change at *p* < 0.05 with respect to the Control group. b: significant change at *p* < 0.05 with respect to the CCl_4_ group.

To study the protective effect of strawberry juice in rats, the modulation of antioxidant defense system was examined, including the activity of SOD, CAT, and GPx enzymes. As shown in Figure 5, CCl_4_ administration led to the modulation of the antioxidant enzymes relative to the control rats. After CCl_4_ administration, SOD, CAT, and GPx activities in the liver homogenates decreased significantly (*p* < 0.05) when compared to the control. On the other hand, strawberry juice supplementation elevated the activities of SOD, CAT, and GPx significantly (*p* < 0.05) compared to the CCl_4_ group. Consistent with the biochemical findings, a significant down-regulation in the SOD2, CAT, and GPx1 gene expression levels caused by CCl_4_ was observed when compared to the control group. In contrast, the strawberry juice treatment significantly up-regulated the SOD2, CAT, and GPx1 gene expression levels (Figure 6).

#### Histopathologic findings

**Figure 7:**
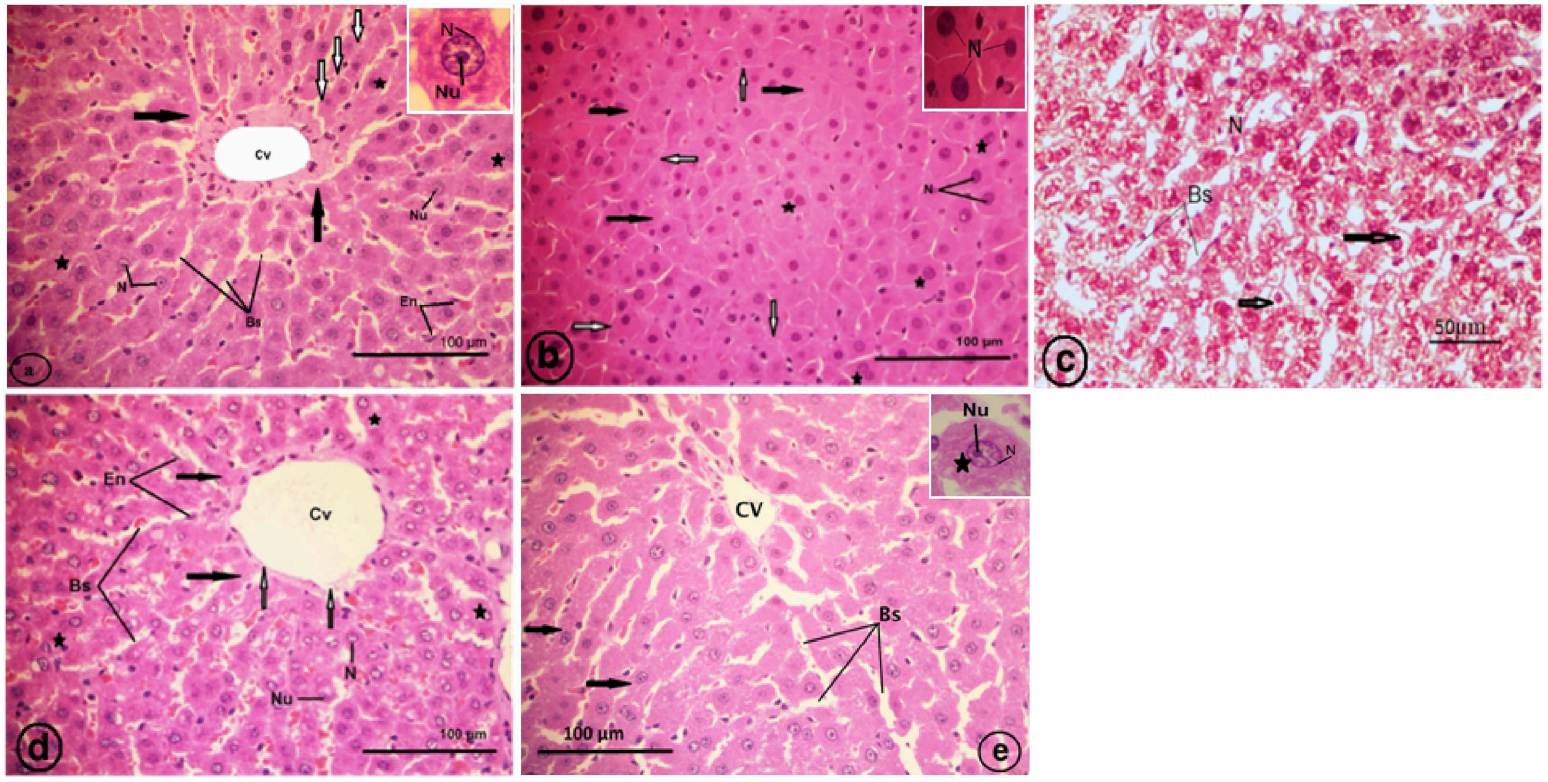
**Light micrographs showing a liver section of rat (Two histological slides from each block from each rat were stained with hematoxylin and eosin; *n* = 10). (A)** Control liver showing no abnormality, the liver section showing a classic hepatic strands (white arrows) with hepatocytes (asterisk) of control rat separated by blood sinusoids (Bs); central vein (Cv); endothelial cell (En) of blood sinusoid. Inset: a hepatocyte with finely granular cytoplasm (black arrows); euchromatic nucleus (N) and prominent nucleolus (Nu). **(B)** CCl_4_ treated group, showing degranulation of hepatocytes cytoplasm (black arrows); collapsed blood sinusoids (white arrows); an increase of binucleated hepatocytes (asterisks). Inset: pyknotic nuclei (N) of hepatocytes. **(C)** CCl_4_ treated group, showing vacuolated narrow irregular strands of hepatocytes (arrows) with compressed cytoplasm against plasma membranes, clumped outside the nucleus (N); blood sinusoids (Bs). **(D)** Showing classical hepatic plates (black arrows) of strawberry administrated group; blood sinusoids (Bs); central vein (Cv); endothelial cell (En) of sinusoids; hepatocytes (asterisks) with finely granular cytoplasm, euchromatic nuclei (N) and prominent nucleoli (Nu). **(E)** Showing recovery of the hepatic plates (arrows) of CCl_4_ and strawberry treated group separated by blood sinusoids (Bs); central vein (Cv).

Normal histological architecture was observed in the liver sections of the control rats (Figure 7A). The CCl_4_-treated group (CCl_4_), revealed an obliteration of the architecture of the hepatic strands, degranulation of the cytoplasm of hepatocytes, collapsed blood sinusoids, an increase of binucleated hepatocytes, and vacuolated narrow irregular strands of hepatocytes with compressed cytoplasm against the plasma membranes and clumped outside the nucleus (Figures 7B,C). The liver of the strawberry supplemented rats (Strb) exhibited classical hepatic plates with finely granulared cytoplasm, euchromatic nuclei, and prominent nucleoli (Figure 7D). Interestingly, the strawberry and CCl_4_ treated group (Strb+CCl_4_) revealed a recovery of the hepatic tissue (Figure 7E).

**Figure 8:**
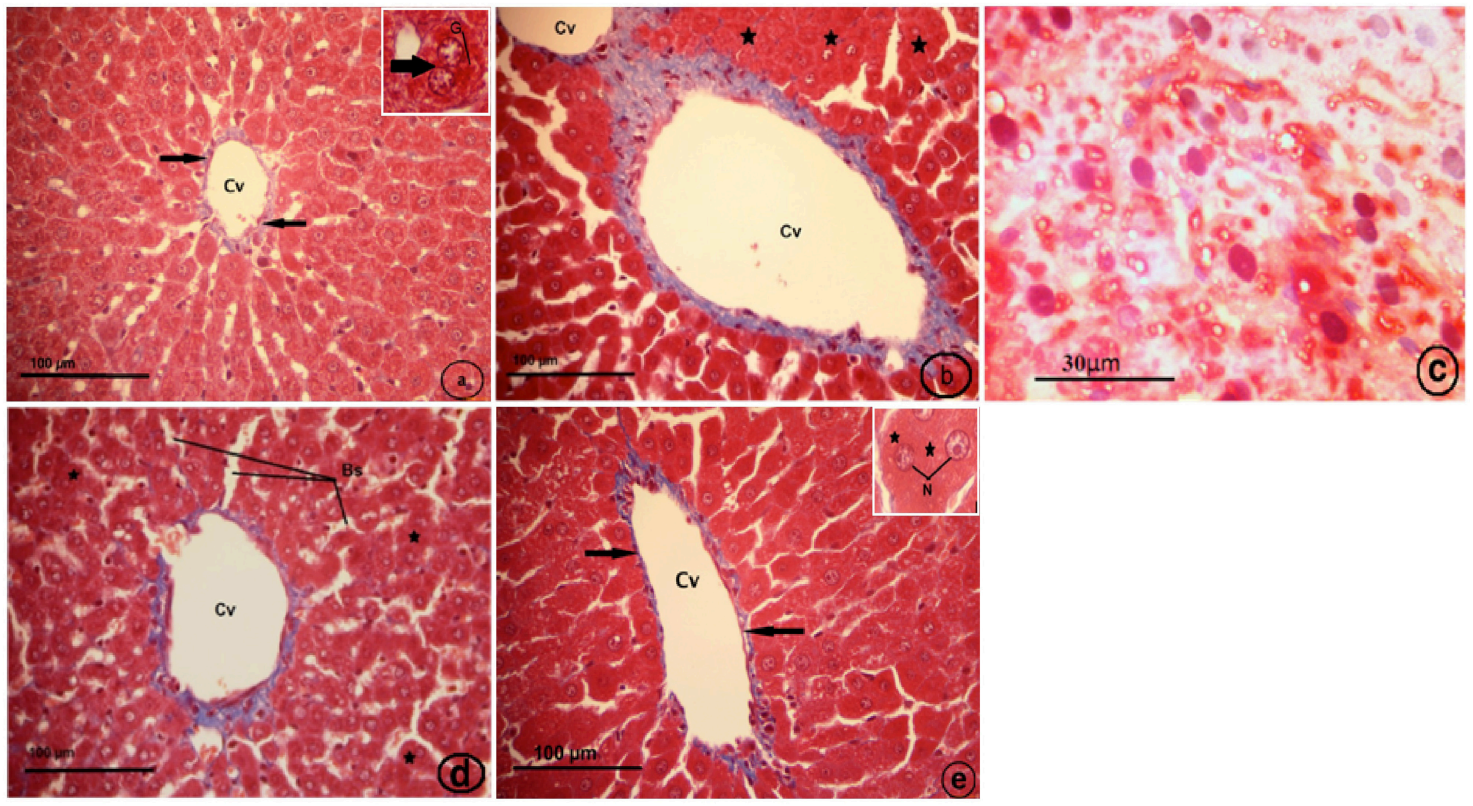
**Light micrographs showing a liver section of rat (Two histological slides from each block from each rat were stained with Masson's trichrome; *n* = 10). (A)** control liver showing very thin blue color of collagen fibers surrounding the central vein (Cv) (black arrows). Inset: binuclear hepatocyte (arrow), glycogen (G). **(B)** CCl_4_ treated group, showing fibrosis in which extended collagen fibers (blue color) extended between two adjacent central veins (Cv), collapsed blood sinusoid (asterisk). **(C)** CCl_4_ treated group, showing fatty liver; hepatocytes with pyknotic nuclei (N).Notice, no distinct cell boundaries. **(D)** Strawberry treated group, showing hepatocytes with intense acidophilic cytoplasm (asterisk), and normal distribution of blue color collagen fibers around central vein (Cv) more or less similar to control; blood sinusoids (Bs). **(E)** Showing recovery of hepatic tissue of CCl_4_ and strawberry treated group. Notice, a faint thin blue layer of collagen fibers (black arrows) around the central vein (Cv). Inset: hepatocytes with euchromatic nuclei (N) and granular cytoplasm.

Histological examination of the control liver sections stained with Masson's trichrome showed very thin blue colors of collagen fibers surrounding the central vein (Figure 8A). The CCl_4_-treated group (CCl_4_), displayed fibrosis in which collagen fibers extended between the two adjacent central veins, collapsed blood sinusoid (Figure 8B), and the fatty liver (Figure 8C). The strawberry treated group (Strb), showed hepatocytes with intense acidophilic cytoplasm and a normal distribution of blue color collagen fibers around the central vein, which is similar to the control (Figure 8D). The CCl_4_ and strawberry-treated group (Strb+CCl_4_) resulted in a recovery of the hepatic tissue, with a faint thin blue layer of collagen fibers around the central vein (Figure 8E).

#### Effect of CCl_4_ on apoptotic markers in the liver

**Figure 9:**
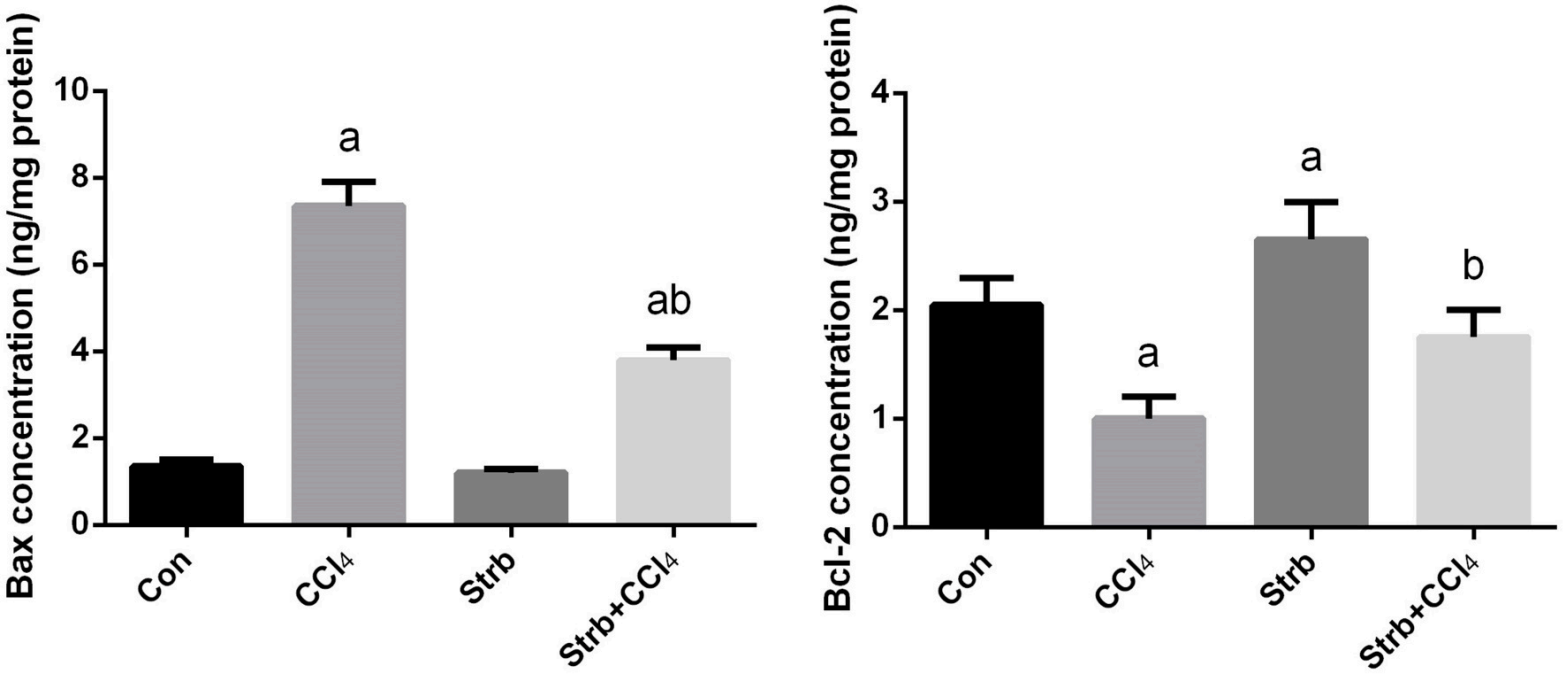
**Effect of strawberry juice supplementation for 12 weeks on hepatic anti-apoptotic/pro-apoptotic protein levels measured by ELISA method in experimental groups**. Values are means ± SEM (*n* = 10). a: significant change at *p* < 0.05 with respect to the Control group. b: significant change at *p* < 0.05 with respect to the CCl_4_ group. Bax, BCL2-Associated X Protein; Bcl-2, B-cell lymphoma 2.

**Figure 10:**
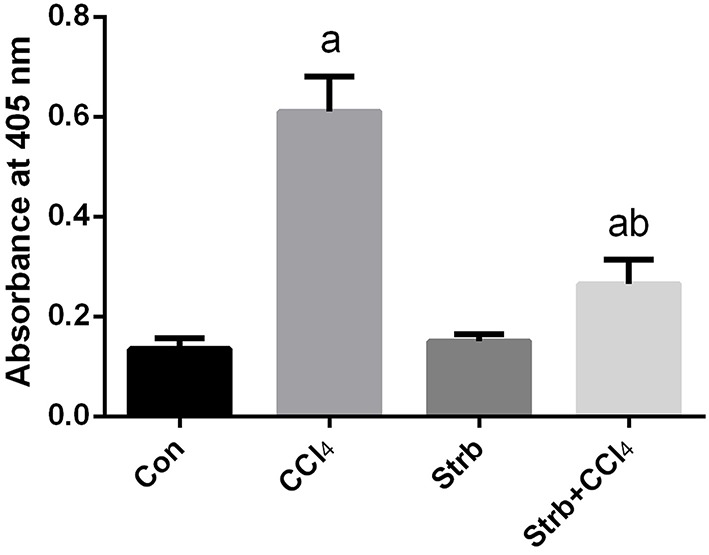
**Effect of strawberry juice supplementation for 12 weeks on the activity of caspase-3-like proteases in liver lysates of rats treated with CCl_4_ (2 ml CCl_4_\kg body weight) for 10 weeks**. Data are expressed as the increase in absorbance at 405 nm due to the release of p-nitroanilide (*n* = 10). a: significant change at *p* < 0.05 with respect to the Control group. b: significant change at *p* < 0.05 with respect to the CCl_4_ group.

To study the anti-apoptotic effects of strawberry juice supplementation in rats, Bax, Bcl-2, and caspase-3 were measured in the liver homogenate. As shown in Figures 9, 10, the levels of Bax and caspase-3 in the CCl_4_-treated rats were higher than that in the control (*p* < 0.05). Supplementation with strawberry juice decreased the Bax and caspase-3 levels significantly compared to the CCl_4_-treated rats. As shown in the present data, Bax was minimally present, whereas Bcl-2 was constitutively expressed in liver tissue. Significant (*p* < 0.05) elevation in level of Bcl-2 was observed in the CCl_4_ and strawberry-treated rats when compared with the CCl_4_-treated group.

## Discussion

Hepatotoxicity induced by CCl_4_ is the most commonly used model system for the screening of hepatoprotective activity of plant extracts/drugs. The oxidative stress induced by CCl_4_, an established model for the evaluation of hepatotoxicity (Yun et al., 2009; Adesanoye and Farombi, 2010; Coballase-Urrutia et al., 2011), was manifested in the hepatic injury observed in the animals treated with CCl_4_. This is due to the CYP P450-mediated metabolism of CCl_4_ to reactive metabolites: CCl_3_· and CCl_3_OO·. These radicals bind irreversibly to cellular molecules such as nucleic acids, proteins, and lipids, especially the polyunsaturated fatty acids to initiate a process of lipid peroxidation by attacking the methylene bridges of unsaturated fatty acid side chains. This invariably affects the mitochondrial permeability, endoplasmic sequestration, and homeostasis and ultimately leads to cell damage (Boll et al., 2001). Our data show that the treatment with CCl_4_ at a dose of 2 ml/kg body weight once per week for 10 weeks led to the development of hepatic injury and fibrosis in rats. The results obtained in this work are similar to the findings of Ganie et al. (2013) and Breikaa et al. (2013), who have reported the hepatotoxic effects of CCl_4_. As a result of hepatic injury, the administration of CCl_4_ exerts possible hepatotoxicity as verified by the elevation in serum ALT and AST activities. In fact, these enzymes are known as important markers of hepatocellular damage as affirmed by Dkhil et al. (2014). This damage was in accordance with the cellular damages and loss of hepatic tissue structural pattern in CCl_4_-treated animals (Figure 7). In consistent, Atawia et al. (2016) demonstrated that increase of serum ALT and AST activities is a constant finding following CCl4 treatment in different experimental animal models. ALT and AST are cytoplasmic aminotransferases that release extracellularly and go into the circulation upon hepatocytes damage. However, strawberry juice supplementation strongly decreased transaminases enzyme in the serum. These findings were consistent with those of many previous studies that showed administration of several antioxidant mixtures or herbal medicines significantly declined the levels of serum marker enzymes in CCl_4_ treated animals as mentioned by Go et al. (2016).

Lipid peroxidation has been implicated in the pathogenesis of hepatic injury by the free radical derivatives of CCl_4_ and is responsible for the cell membrane damage and consequent release of marker enzymes indicating of hepatotoxicity (Danni et al., 1991). In the present study, the significantly elevated levels of TBARS, the products of membrane lipid peroxidation, observed in CCl_4_ administered rats indicated hepatic damage. The CCl_4_ metabolites react with polyunsaturated fatty acids and form covalent adducts with lipids and proteins. These events led to lipid peroxidation and the destruction of cell membranes with consequent liver injury (Szymonik-Lesiuk et al., 2003). Treatment of strawberry juice prevented lipid peroxidation, which could be attributed to the radical scavenging antioxidant constituents (Yuan et al., 2008). The antioxidant effect of flavonoids found in strawberry juice enhanced the process of regeneration. This result might be due to the destruction of free radicals, supplying a competitive substrate for unsaturated lipids in the membrane and/or accelerating the repair mechanism of the damaged cell membrane.

Oxidative stress induced by CCl4 exposure can regulate numerous redox-sensitive transcription factors such as nuclear factor-κB (NF-κB). NF-κB activation was tightly associated with the release of pro-inflammatory cytokines including nitric oxide. CCl_4_-treated animals exhibited a significant elevation in nitrite/nitrate concentration a marker of nitric oxide; however, these levels were inhibited by the strawberry juice. Increased nitrite/nitrate production would be attributed to NF-κB-induced inducible nitric oxide synthase expression following CCl_4_ administration (Atawia et al., 2016). Nitric oxide is involved in pathogenesis of acute hepatotoxicity mainly through depletion of hepatic GSH and inhibition of antioxidant enzymes as well as generation of the highly toxic derivative, peroxynitrite, through reaction with free superoxide.

GSH is the main antioxidant found in liver cells, and it plays a protective role in the metabolism of a large number of toxic agents and preserves cytochrome P450 by blocking lipid peroxidation (Al-Olayan et al., 2014). In the present study, strawberry juice markedly increased the hepatic-maintained GSH level even after the treatment of CCl_4_. CCl_4_ administration led to a significant decrease in the GSH level which can be an important factor in the CCl_4_ hepatotoxicity. The mechanism of hepatoprotection by strawberry juice against CCl_4_ toxicity may be due to the restoration of the GSH level. Among the cellular antioxidants, SOD catalyzes the dismutation of superoxide anion to H_2_O_2_ and O_2_, while, catalase decomposes H_2_O_2_ to water. Several studies have suggested that the phytochemical content and antioxidant/free radical scavenging effect of fruits and vegetables contributes to their protective effect against chronic and degenerative diseases (Heinonen et al., 1998). Strawberry extracts were found to have higher antioxidant activity than extracts from other studied fruits. However, vitamin C is not the only contributor to the antioxidant activity of fruits and vegetables. The antioxidant properties of strawberries have been shown to be mainly due to the high content of phenolic compounds (Heinonen et al., 1998; Vinson et al., 2001).

The impairment of the antioxidants defense system is a critical step in CCl_4_-induced injury. At the gene expression level, the current findings indicated that, CCl_4_ induced down-regulation of the gene expression of all examined antioxidant enzymes (CAT, SOD2, and GPx1). Similar results were obtained in the CCl_4_ induced liver fibrosis in mice (Chen et al., 2013). The present findings also suggest that, strawberry juice augmented the antioxidant status via up-regulation of CAT, SOD2, and GPx1 expression.

We further tried to evaluate the strawberry juice hepatoprotection and show whether it attenuated oxidative stress or inhibited fibrosis in rats treated with CCl_4_. We found that, after the supplementation of strawberry juice, liver tissue showed a normal lobular pattern with a mild degree of necrosis and a lymphocytic infiltration almost comparable to the normal control. Histological investigations revealed that CCl_4_ exposure caused progressive alterations in the liver regions. The findings corroborated well with Bhadauria (2012) and Abdel Moneim (2012) in the fact that CCl_4_ has been implicated in the pathogenesis of several clinical disorders. Strawberry juice supplementation could improve the altered liver histopathologies to some extent.

Additionally, in our study the supplementation of strawberry juice also led to a decrease in the degree of CCl_4_-induced necrotic cell death and protected the liver from the adverse effect of the toxin, this is in accordance with a previous report by Manna et al. (2011). The strawberry scavenges oxygen and nitrogen-based reactants generated in the mitochondria, stabilizes the mitochondrial membrane and enhances the anti-apoptotic signaling. To explore the mechanism of strawberry juice on the attenuation of CCl_4_-induced apoptosis, the amount of caspase-3, Bax and Bcl-2 was measured in liver homogenates. ROS have been shown to increase the permeability of the mitochondrial membrane, resulting in mitochondrial failure (Huang et al., 2008). The permeability of the mitochondrial membrane is dependent upon the transition pore resulting from the release of cytochrome *c* from the mitochondria to the cytosol (Yang et al., 2014). Once released, cytochrome *c* can bind to Apaf-1 in the cytoplasm forming a complex that can activate caspase-9 with subsequent activation of death-inducing caspase-3 (Ghribi et al., 2002). In our study, we observed CCl_4_-induced apoptosis in the liver of rats.

The mitochondria-mediated intrinsic pathway is controlled by Bcl-2 family proteins. The Bcl-2 protein family is classified into two subgroups according to structural homology; the anti-apoptotic proteins such as Bcl-2 and Bcl-XL, and the pro-apoptotic proteins such as Bax and Bak. The balance between the pro- and anti-apoptotic proteins of the Bcl-2 family is important to determine cell survival and death. Bcl-2 may function as a counteracting force to prevent damage by reducing lipid peroxidation triggered by cytotoxic stimuli such as ROS (Akifusa et al., 2009; Abdel Moneim, 2016). Bcl-2 was also found to prevent the release of cytochrome *c*. In contrast, Bax regulates apoptosis, not only by dimerizing with anti-apoptotic Bcl-2 proteins, but also by regulating cytochrome *c* release and subsequent caspase-3 activation (Cai et al., 2011). Our results showed that rats treated with strawberry juice reversed the alternations of Bcl-2 and Bax levels induced by CCl_4_, and substantially restored the ratio of Bcl-2/Bax. In the current report, strawberry juice inhibited all toxic events induced by CCl_4_. It is recognized that strawberry juice scavenges oxygen and nitrogen-based reactants generated in the mitochondria, stabilizes the mitochondrial membrane, and enhances anti-apoptotic signaling.

Plants are well-known and promising prospects for the discovery of new therapeutic products. In recent years, ample interest has been expressed in finding natural antioxidants from commonly available wild plants, fruits and vegetables that were generally mistreated (Abdel Moneim, 2013). Phenolic compounds possess diverse biological activities and are thought to be beneficial for treating oxidative stress induced cell damage. These activities might be related to their antioxidant activity because of their ability to scavenge free radicals by virtue of the presence of hydroxyl groups (Djeridane et al., 2006). It can be stated that free radical scavenging effect is not limited to phenolic compounds but is also from the presence of other antioxidant secondary metabolites in the extracts that directly or indirectly contribute to the activity. The antioxidant properties of phenolic phytochemicals have been reported to exert short-term protection against oxidative stress and related diseases. In addition, polyphenols are known transcriptional regulators, thus mediating long-term effects (Abou-Agag et al., 2001).

The antioxidants play an important role in the regulation and maintenance of metabolism in the body against oxidative stress. It was reported that the induction of antioxidant enzymes reflected an enhancement in cellular protection, ensuring that potential oxidants are metabolized and therefore eliminated more rapidly (Chang et al., 2008; Abdel Moneim, 2014). However, polyphenols are known to modulate the transcription and expression of proteins related to the endogenous antioxidant defense. They do so by interacting with antioxidant response elements in gene promoter regions of genes that encode proteins related to oxidative injury management (Moskaug et al., 2005). Differential display is considered one of the most recently valuable molecular techniques. It is able to provide researchers with a general overview of the down and up-expressed genes under specific treatments without previous knowledge about the extent of their expression or identity. In our study, we used differential display to compare between the genetic status of the liver tissues in normal and treated conditions.

Our findings demonstrated that the oxidative stress elicited by CCl_4_ intoxication has been nullified due to the protective effect of strawberry juice and the findings indicate that strawberry juice possessed antioxidant, anti-apoptotic and anti-fibrotic properties, probably mediated by the presence of polyphenols and flavonoids compounds.

## Author contributions

ME, SSH, NA, SBH, EA, AA, ZH, RA made a significant contribution to conception and design of the study, acquisition and analyses of data and drafting of the manuscript and the revised manuscript.

### Conflict of interest statement

The authors declare that the research was conducted in the absence of any commercial or financial relationships that could be construed as a potential conflict of interest.
